# A cost-analysis of conducting population-based prevalence surveys for the validation of the elimination of trachoma as a public health problem in Amhara, Ethiopia

**DOI:** 10.1371/journal.pntd.0008401

**Published:** 2020-09-03

**Authors:** Randall P. Slaven, Aisha E. P. Stewart, Mulat Zerihun, Eshetu Sata, Tigist Astale, Berhanu Melak, Melsew Chanyalew, Demelash Gessese, Paul M. Emerson, Zerihun Tadesse, E. Kelly Callahan, Scott D. Nash, Deborah A. McFarland

**Affiliations:** 1 The Carter Center, Atlanta, Georgia, United States of America; 2 The Carter Center, Addis Ababa, Ethiopia; 3 Amhara Regional Health Bureau, Bahir Dar, Amhara, Ethiopia; 4 International Trachoma Initiative, Decatur, Georgia, United States of America; 5 Hubert Department of Global Health, Rollins School of Public Health, Emory University, Atlanta, Georgia, United States of America; RTI International, UNITED STATES

## Abstract

**Background:**

Trachoma prevalence surveys, including impact surveys (TIS) and surveillance surveys (TSS), provide information to program managers on the impact of the SAFE (surgery, antibiotics, facial cleanliness, and environmental improvement) strategy and current burden of disease, and they provide a crucial component of the evidence base necessary for the validation of the elimination of trachoma as a public health problem. The prevalence surveys included in this analysis are multi-level cluster random surveys that provide population-based estimates for program planning. This study conducted an analysis of the cost of 8 rounds of TIS/TSS executed in Amhara, Ethiopia, 2012–2016, comprising 232,357 people examined over 1,828 clusters in 187 districts.

**Methodology and findings:**

Cost data were collected retrospectively from accounting and procurement records from the implementing partner, The Carter Center, and coded by survey activity (i.e. training and field work) and input category (i.e. personnel, transportation, supplies, venue rental, and other). Estimates of staff time were obtained from The Carter Center Ethiopia. Data were analyzed by activity and input category. The mean total cost per cluster surveyed was $752 (standard deviation $101). Primary cost drivers were personnel (39.6%) and transportation (49.2%), with costs increasing in the last 3 rounds of TIS/TSS.

**Conclusion:**

Despite the considerable cost of conducting TIS and TSS, these surveys provide necessary information for program managers. Limited options are available to reduce the costs of TIS/TSS and gain economies of scale, as the surveys must be designed to achieve their designated sample size. However, surveys must also be designed in a way that is possible to be executed given the financial resources, personnel, and time required. Program managers can use these findings to improve estimates of the total cost of a survey and its components to ensure that sufficient resources are budgeted accordingly.

## Author summary

Population-based trachoma surveys are necessary to determine the impact of interventions and to build the case for the validation of elimination of trachoma as a public health problem. Many trachoma-endemic areas are currently receiving mass drug administration, which will require trachoma impact surveys (TIS) and trachoma surveillance surveys (TSS). We analyzed the costs accrued by the implementing non-governmental organization during 8 rounds of trachoma surveys in Amhara, Ethiopia, that included 187 districts surveyed over four years. The costs were sorted by activity (i.e. training and field work) and input category (i.e. transportation, personnel, venue rental, supplies, and other). Field work was the most expensive activity for trachoma surveys, with transportation and personnel as the most significant drivers of cost. As the global trachoma program matures, these surveys must be executed given the financial resources, personnel, and time available. Program managers can use these findings to improve total cost estimates of trachoma surveys and survey components to ensure that sufficient resources are being budgeted accordingly. Additionally, managers in hyperendemic settings can use these findings to better understand the drivers in cost of repeated surveys in a single geography.

## Introduction

Trachoma, a neglected tropical disease, is one of the leading causes of infectious blindness worldwide [1]. By 2020, World Health Assembly resolution 51.11 has scheduled trachoma for global elimination as a public health problem [2]. In order to meet World Health Organization (WHO) requirements for the validation of elimination of trachoma as a public health problem, national programs must submit evidence to show that each formerly endemic district, normally defined as an administrative unit for health care management, has a prevalence of trachomatous trichiasis (TT) unknown to the health system of <1 case per 1,000 total population, and a prevalence of trachomatous inflammation-follicular (TF) among children aged 1–9 years of <5% [3].

Population-based prevalence surveys are the preferred methodology to estimate the prevalence of trachoma in a given population. With these surveys a multi-stage sampling method is used, whereby clusters in eligible enumeration units are selected in the first stage and households within clusters are selected in the second stage. All people living in selected households are eligible to be examined for the clinical signs of trachoma using the WHO Simplified Grading System [4]. Trachoma impact surveys (TIS) are used to monitor districts for the impact of the SAFE (surgery, antibiotics, facial cleanliness, and environmental improvement) strategy on the prevalence of TF among children aged 1 to 9 years after several years of intervention and thus whether continued interventions are required [5]. Trachoma surveillance surveys (TSS) use similar methodology and are conducted at least 2 years after the last TIS in order to assess for reemergence of TF within this age range. Trachoma programs are fully reliant on these surveys for their ongoing monitoring and evaluation efforts, and the results of these surveys are required in order to continue to receive donated antibiotic. As programs mature, managing the cost of these surveys on an ongoing basis will be important. This paper lays out detailed longitudinal TIS and TSS cost data from a long-running regional trachoma control program in Ethiopia, an important country context for international trachoma elimination efforts. Given the number of TIS/TSS surveys that will be required to build the evidence base necessary to validate the elimination of trachoma as a public health problem world-wide, it is vital to better understand the ongoing costs of these surveys.

Between 2012 and 2016 the Trachoma Control Program in Amhara, Ethiopia conducted 8 biannual rounds of TIS/TSS, which encompassed 187 district-level surveys, such that several of the 152 districts in the region were surveyed at least twice. This study analyzed the costs incurred by a non-governmental organization (NGO) in conducting these surveys over 4 years in a hyperendemic region of Ethiopia.

## Methods

### Ethical considerations

Survey methodology was approved by the ethical review committee of the Amhara Public Health Research Institute, Ethiopia and Emory University Institutional Review Board (protocol 079–2006). Permission to obtain verbal informed consent and assent was granted by the review boards due to the high rate of illiteracy among the study population.

### Data collection

Data on the location, number of districts, number of clusters, dates of survey execution, and number of survey teams for 8 biannual rounds of TIS/TSS were compiled from existing Carter Center Ethiopia program records and verified with published reports. All data came from district-level surveys, spanning 1,828 clusters surveyed in a total of 187 districts. Districts are locally called ‘*woredas’* and are the administrative unit for health care in the region. A cluster was defined as a group of households within a ‘*gott*’ or village and the geographic area that they covered as selected by a probability proportional to estimated size method [6, 7]. The sampling frame included all villages in the district regardless of ease of access by survey teams. Data were collected electronically using a bespoke software package on Android tablets[8].

To capture the expense of the 8 rounds of TIS/TSS, every cost incurred by The Carter Center in Ethiopia from January 2012 through February 2017 (n = 36,840) was exported from accounting records using Intuit’s Quickbooks (Intuit, Enterprise Canada version, Mountain View, California, 2016). All costs were exported to Microsoft Excel 2016 and reviewed line by line to isolate all TIS/TSS-related expenses for further coding. Multiple checks were used to conduct the review, which was executed twice to ensure costs were not missed or mis-coded. Additional costs incurred in Atlanta, where The Carter Center is based, for supplies used in TIS/TSS were gathered from procurement records and were added to the data set. All transactions from the final round of TIS/TSS were reviewed by the Carter Center Ethiopia program staff to ensure they were related to TIS/TSS. Receipts from a selection of the 10 largest invoices for the final round of TIS/TSS were also reviewed to ensure applicability and accuracy of coding.

Carter Center staff time was included in the data set, as additional staff would have to be hired or contracted if additional TIS/TSS were to be conducted. The Carter Center Ethiopia program staff provided the number of days they worked on each activity (i.e. training and field work) to perform the most recent round of TIS/TSS, which took place between August and November 2016. Estimates of time spent by staff on earlier TIS/TSS rounds were not requested, as it was assumed that the allocation of effort would be similar and that responses for previous rounds would become increasingly less accurate due to recall bias and attrition [9]. The majority of the staff involved in TIS/TSS in 2016 also worked on previous TIS/TSS. A per cluster cost of staff time was estimated by multiplying each staff member’s daily salary rate (inclusive of benefits) by the reported number of days they spent on each activity for TIS/TSS round 8, which was then divided by the number of clusters surveyed in that round. Estimated compensation costs were added to each of the other TIS/TSS rounds by multiplying the number of clusters in a TIS/TSS by the per cluster rate derived from TIS/TSS round 8 and adding this product to the personnel categories of each of the remaining rounds of TIS/TSS. Round 8 of TIS/TSS took an estimated 491 person-days to conduct surveys in 30 districts (16.4 person-days per district.)

### Data coding

Data were coded in a 3-tiered system for simplicity and comparability based on a review of the literature [10, 11], survey protocols, budgets, and interviews with staff. All expenses were coded as being related to the training or to field work (the Activity), which are the 2 main components of TIS/TSS (Table 1). Training is conducted before each round of surveys to ensure survey teams maintain adherence to the protocols. Costs related to the supervision of training or field work were included in each section to better represent the true cost of each activity. Data were further divided into input categories, which were defined as the primary components of each activity (personnel, transportation, supplies, and venue rental/other). “Other” costs included photocopying, and medical reimbursement. To facilitate comparability with other studies, the Venue Rental input category was originally named “Other”, but was subsequently changed after a review of the expenses indicated only venue rental expenses were present. To enable a more in-depth review of the data, 46 cost codes were defined based on a literature review and review of the most common charges (e.g. transportation costs incurred during field work had codes for vehicle rental, fuel cost, vehicle repair, and driver per diems.)

**Table 1:** Activities and input categories for trachoma impact and surveillance surveys, Amhara, Ethiopia.

	Activities	
	Training	Field Work
**Input Categories**	Personnel	Personnel
	Transportation	Transportation
	Supplies	Supplies
	Venue Rental	Other

Fixed costs, such as building rental or vehicle purchase, were intentionally excluded, as it is assumed that programs conducting these surveys already have these items and that increased TIS/TSS would not result in an increase in these costs. The cost of technical and logistical assistance from headquarters in Atlanta, including salaries or any other headquarters overhead, were not included in the data to improve comparability with other research [10–13] and to simplify the analysis by reducing the number of assumptions about the allocation of these costs to each survey.

After all TIS/TSS costs were compiled in Excel, each was given a numerical code that corresponded to the respective activity and input category. Each expense was also assigned a code that indicated for which of the rounds of TIS/TSS it was incurred, which was derived using the date and location (East or West Amhara sub-region) of that cost as entered into the costing system. A number of observations included expenses for both training and field work and thus could not be accurately allocated between the two activities. In these circumstances, the entire expense was categorized under field work.

Special attention was given to costs incurred by the program that were additive to the surveys but would not normally be part of a TIS/TSS, such as swab collection to analyze ocular *Chlamydia trachomatis* [14] and stool collection to determine the prevalence of intestinal parasites [15, 16]. These costs were removed from the analysis. Specific TIS/TSS-related procurement costs that were accrued at the headquarters level in Atlanta, such as electronic tablets, memory cards, or labels, were provided by the procurement team, coded using the same methodology as the expenses incurred in Ethiopia, and added to the overall data set.

### Data analysis

All costs were normalized to 2016 United States (US) dollars, using the annual gross domestic product (GDP) implicit price deflator for Ethiopia for the year each survey was primarily conducted [17]. Microsoft Excel 2016 was used to generate descriptive statistics on the cost of each round of TIS/TSS, including cost per cluster by activity and input category. Means and standard deviations (SD) were calculated. Because substantial cost differences between early and later survey rounds were detected during the analysis, we also compared costs between the first 5 and the last 3 rounds of surveys using the Student’s t-test.

## Results

The 8 rounds of TIS/TSS included 1,828 total clusters in 187 districts with 232,357 people examined for trachoma. The mean cost per cluster was $752 (SD ± $101) (Table 2). Mean per cluster cost was $686 (± $52) for the first 5 surveys, which was statistically significantly lower than the mean per cluster cost of the final 3 rounds of surveys $863 (± $54), representing a 25.8% increase (P <0.01). The mean per cluster cost of training over 8 rounds was $103 (± $26) and the mean per cluster cost of field work over those 8 rounds was $650 (± $92). The mean per cluster cost of field work increased significantly between the first 5 rounds and final 3 rounds from $589 (± $37) to $752 (± $60), a 27.7% increase (P < .01). The 4 major cost drivers were personnel and venue rental during training and personnel and transportation during field work (Table 3).

**Table 2:** Trachoma impact and surveillance surveys costs, Amhara, Ethiopia, 2012–2016.

Survey Round	Survey 1	Survey 2	Survey 3	Survey 4	Survey 5	Survey 6	Survey 7	Survey 8	All Surveys
Survey Location	East Amhara	West Amhara	East Amhara	West Amhara	East Amhara	West Amhara	East Amhara	West Amhara	West/East Amhara
Survey Date	Dec 2012—Jan 2013	Jun—July 2013	Jan 2014	Jun 2014	Feb 2015	Oct 2015	Dec 2015—Jan 2016	Aug—Nov 2016	Dec 2012—Nov 2016
Clusters, n	317	359	270	119	99	248	128	288	1,828
Districts, n	29	41	33	5	10	26	13	30	187
People Examined, n	34,277	41,294	34,229	15,317	12,510	35,214	17,773	41,751	232,365
Cost Per Cluster, $	724	658	745	598	705	925	792	872	752 *
Cost Per District, $	7,914	5,764	6,097	14,242	6,975	8,820	7,802	8,374	8,249 *
Cost Per Person Examined, $	6.70	5.72	5.88	4.65	5.58	6.51	5.71	6.02	5.84 *
Total Cost: Training, $	31,087	20,019	33,913	8,326	13,669	24,982	14,872	33,613	180,482
Total Cost: Field Work, $	198,424	216,319	167,296	62,884	56,079	204,340	86,555	217,596	1,209,493
**Training** Cost Per Cluster, $	98	56	126	70	138	101	116	117	103 *
**Field Work** Cost Per Cluster, $	626	603	620	528	566	824	676	756	650 *

*Mean of all surveys

**Table 3:** Total costs of trachoma impact and surveillance surveys by activity and input, Amhara, Ethiopia, 2012–2016.

Survey Round	Survey 1	Survey 2	Survey 3	Survey 4	Survey 5	Survey 6	Survey 7	Survey 8
Location of Survey	East Amhara	West Amhara	East Amhara	West Amhara	East Amhara	West Amhara	East Amhara	West Amhara
Date of Survey	Dec 2012—Jan 2013	Jun—July 2013	Jan 2014	Jun 2014	Feb 2015	Oct 2015	Dec 2015—Jan 2016	Aug—Nov 2016
Clusters, n	317	359	270	119	99	248	128	288
Districts, n	29	41	33	5	10	26	13	30
Training								
Personnel, $	17,383	6,660	18,332	6,397	7,178	9,950	8,459	13,070
Transportation, $	-	1,263	141	-	-	-	-	-
Supplies, $	413	209	-	-	-	338	-	-
Venue Rental, $	13,291	11,887	15,440	1,928	6,492	14,695	6,413	20,543
Field Work								
Personnel, $	85,815	104,794	70,489	22,895	19,808	65,361	24,253	69,800
Transportation, $	108,245	97,839	90,732	37,915	34,117	128,913	58,446	126,066
Supplies, $	4,296	13,686	6,075	2,075	2,137	9,402	3,856	18,594
Other, $	68	-	-	-	17	664	-	3,137

Training costs represented a mean of 13.6% of total costs (Table 4). The main cost drivers for training were personnel and venue rental, accounting for 51.0% and 47.7% of total training costs respectively. Field work costs accounted for a mean of 86.4% of total costs. Personnel and transportation costs represented more than 90% of the cost of field work in each survey. Although the total cost and cost per cluster varied between each survey, the proportion of costs for each of these activities was relatively stable.

**Table 4:** Costs by activity and input as proportion of all costs, Amhara, Ethiopia, 2012–2016.

Activity / Category	All Rounds—Mean (SD)
**Training**	**13.6% (3.3)**
Personnel	**51.0%**
Transportation	**0.8%**
Supplies	**0.5%**
Venue Rental	**47.7%**
**Field Work**	**86.4% (3.3)**
Personnel	**37.2%**
Transportation	**58.0%**
Supplies	**4.6%**
Other	**0.2%**

**Fig 1:**
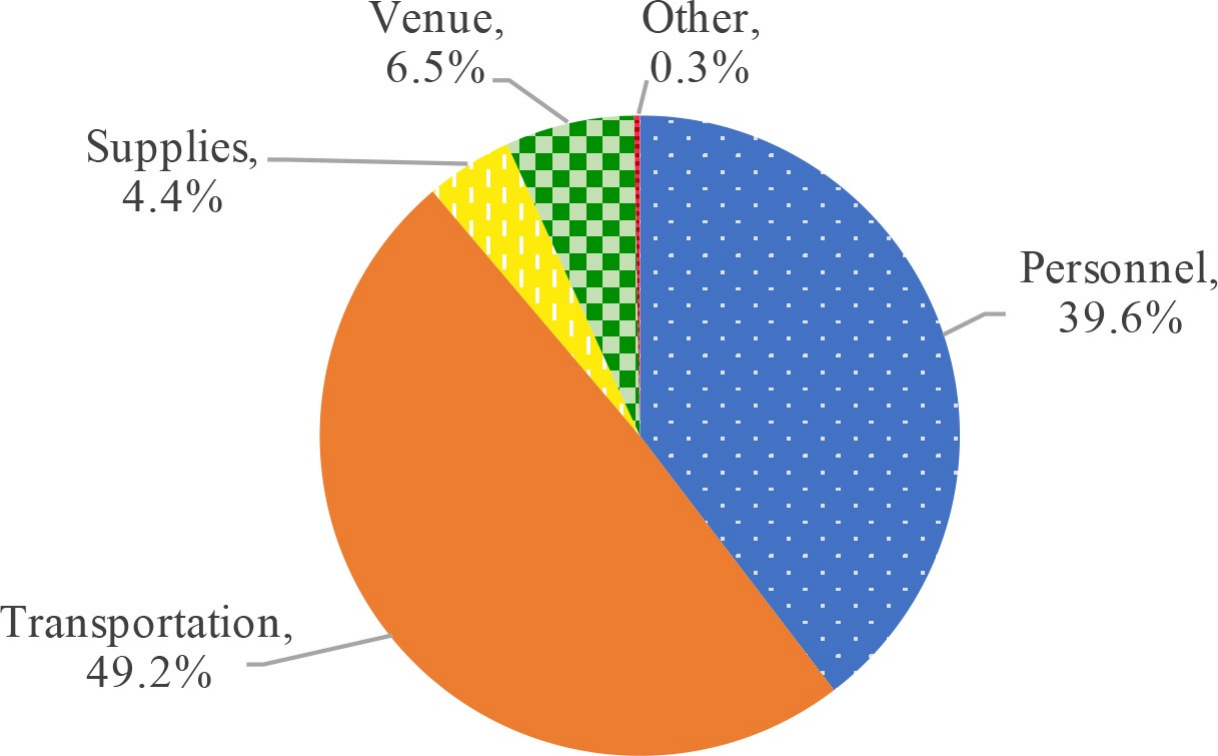
Proportion of total costs by input Category (for all activities) for trachoma impact and surveillance surveys rounds 1–8, Amhara, Ethiopia, 2012–2016.

The transportation and personnel inputs together were responsible for the majority of all cost drivers across both training and field work activities (88.8%) (Fig 1). Per diems represented 79.6% of all personnel costs ($231.97 per cluster), while the derived expense of Carter Center Ethiopia staff time, that were estimated from reports of the effort spent on the 8^th^ round of TIS/TSS, accounts for 20.4% ($59.37 per cluster) of the total personnel cost for all surveys.

**Fig 2:**
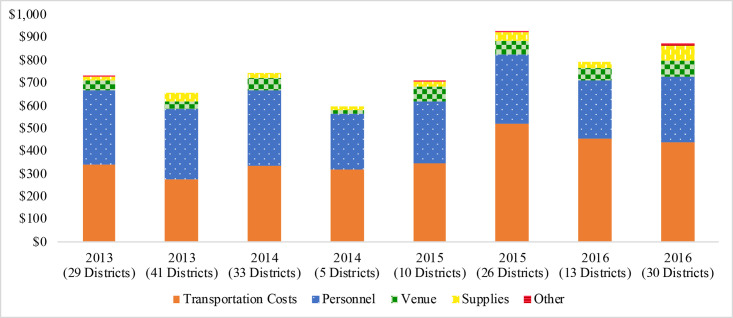
Per cluster costs, by input category for 8 rounds of trachoma impact and surveillance surveys, Amhara, Ethiopia, 2012–2016.

Transportation costs incurred during the survey were the largest single driver of increasing costs over the study time period, especially when the mean per cluster cost of transportation during field work increased to $471 during the final 3 rounds of surveys from $323 for the first 5 rounds of surveys, a 46% increase (Fig 2). The majority of this per cluster increase, 84.0%, was attributable to increases in the cost of transportation during the field work activity (Table 5). The primary driver of the increase in those transportation costs was an increase in vehicle rental costs, which accounted for a $129.77 increase in per cluster cost over the 2 periods (73.3% of the total increase in costs). The proportion of vehicle rentals for all vehicles used did not significantly change between these 2 periods, therefore the increased cost incurred reflects an overall increase in the cost of the input. A slight decrease in per cluster cost of personnel was observed with each subsequent round of surveys costing a mean of $8.28 per cluster less than the previous round.

**Table 5:** Drivers of increased cost from the first 5 rounds to final 3 rounds of impact and surveillance surveys, Amhara, Ethiopia, 2012–2016.

	Mean Per Cluster Cost of First 5 Survey Rounds, $	Mean Per Cluster Cost of Last 3 Survey Rounds, $	Increase (Decrease) from First 5 Survey Rounds to Last 3 Survey Rounds, $	Proportion of Total Increase from First 5 to Last 3 Rounds
**Training**				
Personnel	54	51	(3)	-2%
Transportation	1	-	(1)	0%
Supplies	0	0	0	0%
Venue Rental	43	60	17	10%
**Field Work**				
Personnel	243	232	(11)	-6%
Transportation	323	471	149	84%
Supplies	23	44	22	12%
Other	0	5	4	3%

## Discussion

Comprehensive cost data collected from 187 districts over a 4-year period within a program serving one of the most known trachoma endemic populations in the world has demonstrated that the total per cluster survey cost was $752 per cluster, with a range of $598 to $925 per cluster. Transportation and personnel costs together accounted for the majority of costs, 88.8%. Costs increased significantly for the final 3 rounds of TIS/TSS conducted in the second half of 2015 and 2016 as a result of increased unit costs, as survey methodology did not substantively change over that time period. As the global trachoma program begins reducing its overall treatment targets as a result of successful programming, Ethiopia remains the country with the greatest remaining burden, with 44% of the world’s at-risk population for trachoma[18]. This is despite the presence of mature control programs with several years of at-scale antibiotic distribution [14, 19–21]. As a result, the global program should prepare for significant investment in surveys to ensure the elimination of trachoma as a public health problem, including repeated surveys in the same geographic area, like those analyzed in this paper.

The results of our cost analysis can be compared to those conducted previously in different trachoma contexts. In 2011, The International Coalition for Trachoma Control derived an estimate of the global cost of SAFE and specifically estimated a per district cost of $7,500 for impact surveys [22]. Chen et al. performed an in-depth analysis of trachoma survey costs conducted between 2006 and 2010 in 8 national trachoma control programs [10]. The median cost of a survey was estimated at $4,784, with an IQR of $3,508 to $6,650, per district as observed over 3,203 clusters in 165 districts. The median cost of a cluster was $311 (IQR $119-$393), with clusters per district ranging from 11 to 40. The Global Trachoma Mapping Project estimated a per cluster cost of $692, inclusive of headquarters costs, for mapping trachoma in 16,626 clusters in 1,546 districts across 17 countries [23]. When compared to the cost for Ethiopia alone, however, the cost per cluster ($740) was similar to the estimate we calculated for Amhara. Stelmach et al. conducted an analysis of expenditures on TIS/TSS, finding a median cost per evaluation unit (district) of $8,298 over 322 evaluation units in 11 countries between 2011 and 2018 [24]. Although there are differences in cost categories between ours and previously published studies, we believe our approach was data driven and realistic for how surveys were conducted in this time period. The transparency in our methods should allow other programs to apply our lessons learned to their own budgetary and operational contexts. Based on these estimates, the global trachoma program will require significant annual resources in addition to intervention costs to demonstrate elimination of trachoma as a public health problem.

As of March 2018, the Amhara region had a minimum of 127 districts that would require TIS, and assuming all TIS districts achieved TF <5%, would have a minimum of 158 districts that would require TSS. Using the per cluster estimate of $752 and 30 clusters per district, as of March 2018, Amhara would require a minimum expenditure of $6,429,600 on surveys before successful elimination of trachoma as a public health problem. This estimation likely underestimates the total cost, as many districts will likely require additional rounds of TIS as a result of not meeting elimination targets when surveyed [7, 14, 25]. Assuming half of all districts miss their targets and require an additional TIS, the estimated remaining survey cost escalates to $7,862,160.

Increases in vehicle rental costs were responsible for the majority (73.3% or $130 per cluster) of the cost increase of the final 3 rounds of TIS/TSS. Total vehicle time used for each survey is a function of the number of teams and number of days that the teams are in the field conducting the survey. Attempts to reduce the number of teams may increase the number of days each remaining team spent conducting the survey, resulting in no change to the total days of vehicle time required. The proportion of vehicles which were rented versus owned did not significantly change from the first 5 surveys to the last 3 surveys, suggesting that the cost of renting vehicles over the use of program-owned vehicles was not the cause of this increase and that an increase in the cost of fuel was a more likely driver of increased costs. As this analysis is based on the value of the United States Dollar, certain costs that were budgeted and paid in Ethiopian birr, such as per diem, would have the appearance of reducing over time as the birr weakened against the dollar over the course of the surveys examined. Despite this, future increases in inputs essential to conduct a survey, such as fuel, can dramatically increase the cost per cluster, as evidenced by this study. The slight decrease in personnel costs observed over the 8 survey rounds was likely due to increased efficiency of teams as a result of experience.

Current guidance from the WHO recommends that TIS/TSS be conducted in 20 to 30 clusters per district to detect whether the prevalence of TF among children aged 1 to 9 years is <5% [26]. When considered with the results of this study—that 88.8% of all costs were transportation or personnel—there remains little room for program managers to reduce costs or gain economies of scale costs unless there is a modification in survey methodology to reduce the number of clusters visited. Further research is needed into optimizing cluster survey designs that balance accuracy with cost-efficiency across a range of epidemiological settings [27]. Computer modeling could be an important tool on this front.

Strengths and limitations of this study stem from the methods used to collect cost data. Cost information was aggregated over an entire round of TIS/TSS and did not provide the detail necessary to report on the cost of each district individually, which prevents an analysis of what might cause certain districts to be more or less expensive than others, however, costs were comprehensively gathered, and each district must be surveyed, lowering the utility of district-by-district information. The data were gathered through a detailed, proactive search of all accounting records and did not rely on self-reported or budgeted figures. Additionally, accounting practices did not consistently include information that would enable fuel costs to be divided accurately between training and field work. In such cases, costs were coded as field work. The impact of this lack of specificity in the data was believed to be relatively small, given that significantly less fuel is used during training than field work. Any mis-categorization between field work and training would not have altered the total cost per cluster estimate, and as the surveys reviewed took place over approximately 4 years, the overall consistency in our results suggests the total cost per cluster was not affected by this issue. As part of the study design, we deliberately excluded costs incurred by the implementing NGO to clean or analyze the data, which was necessary to reduce the scope of the data collection and number of assumptions necessary. Similarly, the Ministry of Health perspective was excluded from this analysis. In no way should that exclusion be seen as a minimization of their role or importance in the survey process. Further work may seek to collaborate with governmental ministries to quantify their inputs to take a full accounting of costs associated with this work. Using 2016 for interpolation of salary data might underestimate the costs for the entire period since these data were from a mature program at scale which had been conducting TIS/TSS surveys for several years. It is likely that to train, equip, and execute TIS/TSS for the first time would be costlier than observed in a mature program.

The cost estimates identified by this study were all incurred in the same geographic area by one NGO, and thus are less generalizable than if this study included surveys conducted in other areas by other NGOs; however, Amhara is a large, geographically diverse region that encompasses over 20 million people, thus making it useful for other programs in Ethiopia, the highest trachoma burden country in the world. Future inquiry could gather information from the government as well, to more holistically represent cost. Despite these limitations, this report provided comprehensive cost data within a large trachoma program and allowed for an analysis of the changes in costs over time as well as the drivers of those changing costs. As the prevalence of TF drops in a region on account of interventions, as has been observed in Amhara, the number of required annual surveys will increase. Therefore, understanding how survey costs change over time within a program are important for programs throughout Ethiopia and globally. Future research could investigate the implementation costs of the components of SAFE, as they are costs that trachoma control programs must monitor and budget for.

Despite the considerable cost of conducting TIS and TSS, these surveys remain a vital tool for trachoma programs. These surveys provide actionable data that is required for countries to complete applications for azithromycin donation which saves the country the cost of purchasing this medication. As countries move toward the elimination of trachoma as a public health problem, the evidence provided by TIS/TSS will continue to play an important role in the prioritization of limited programmatic resources and in the ultimate creation of dossiers necessary for national validation of the elimination of blinding trachoma as a public health problem. Program managers and donors can use these results to ensure that sufficient annual resources are devoted to these surveys and that program priorities are established. In resource-challenged areas, it may be difficult for ministries of health to find the funding necessary to conduct these surveys, which would prevent them from building the dossier necessary for the validation of elimination of trachoma as a public health problem.

## Supporting information

S1 Checklist STROBE checklist. (DOC)
