# Supplementary material for: A cost-analysis of conducting population-based prevalence surveys for the validation of the elimination of trachoma as a public health problem in Amhara, Ethiopia
Source: PLoS Negl Trop Dis. 2020 Sep 3;14(9):e0008401. doi: 10.1371/journal.pntd.0008401 (PMC7494078; doi:10.1371/journal.pntd.0008401)
Supplement: S1 Checklist — (DOC) [file pntd.0008401.s001.doc]

S1. STROBE Statement—Checklist of items that should be included in reports of ***cross-sectional studies***

|  | Completed  Yes/ No | Item No | Recommendation |
| --- | --- | --- | --- |
| **Title and abstract** | Yes | 1 | (*a*) Indicate the study’s design with a commonly used term in the title or the abstract  *The title describes the study design as “population based prevlance surveys”* |
|  | Yes |  | (*b*) Provide in the abstract an informative and balanced summary of what was done and what was found  *The abstract describes the methods and findings.* |
|  | Introduction | | |
| Background/rationale | Yes | 2 | Explain the scientific background and rationale for the investigation being reported  *The background and rational are described in the Introduction, paragraphs 1 & 2* |
| Objectives | Yes | 3 | State specific objectives, including any prespecified hypotheses  *The study objective is discussed in paragraph 3 of the Introduction.* |
|  | Methods | | |
| Study design | Yes | 4 | Present key elements of study design early in the paper  *The study design is discussed in the ‘data collection’ sub-section of the methods section* |
| Setting | Yes | 5 | Describe the setting, locations, and relevant dates, including periods of recruitment, exposure, follow-up, and data collection  *The programmatic setting, location and dates of surveys are described in the ‘data collection’ sub-section of the Methods section.* |
| Participants | Yes | 6 | (*a*) Give the eligibility criteria, and the sources and methods of selection of participants  *Selection of sample and eligibility criteria are discussed in the ‘data collection’ sub-section of Methods section* |
| Variables | Yes | 7 | Clearly define all outcomes, exposures, predictors, potential confounders, and effect modifiers. Give diagnostic criteria, if applicable  *Outcomes for survey cost analysis are defined in the ‘data coding’ sub-section of the method section including Table 1..* |
| Data sources/ measurement | Yes | 8* | For each variable of interest, give sources of data and details of methods of assessment (measurement). Describe comparability of assessment methods if there is more than one group  *Survey cost categories for analysis as well as the sources for those data are expalained both in the ‘data collection’ and ‘data coding’ sub sections of the methods ection.* |
| Bias | Yes | 9 | Describe any efforts to address potential sources of bias  *Steps to reduce bias in data collection are addressed in paragraph 2 of ‘data collection’ sub section of Methods, and steps to reduce bias in the analysis are addressed in paragraph 2,3,&4 in ‘data coding’ paragraph.* |
| Study size | Yes | 10 | Explain how the study size was arrived at  *Sample size for this analysis was dependent on the sample available from the original surveys which is described in paragraph 1 of ‘data collection’ sub section of Methods.* |
| Quantitative variables | Yes | 11 | Explain how quantitative variables were handled in the analyses. If applicable, describe which groupings were chosen and why  *Use of variables is dicussed in ‘data analysis’ sub-section of Methods section.* |
| Statistical methods | Yes | 12 | (*a*) Describe all statistical methods, including those used to control for confounding  *Statistical methods discussed in ‘data analysis’ sub-section of Methods section* |
|  | Yes |  | (*b*) Describe any methods used to examine subgroups and interactions  *Statistical methods discussed in ‘data analysis’ sub-section of Methods section* |
|  | Not applicable |  | (*c*) Explain how missing data were addressed |
|  | Not applicable |  | (*d*) If applicable, describe analytical methods taking account of sampling strategy |
|  | Not applicable |  | (*e*) Describe any sensitivity analyses |
|  | Results | | |
| Participants | Yes | 13* | (a) Report numbers of individuals at each stage of study—eg numbers potentially eligible, examined for eligibility, confirmed eligible, included in the study, completing follow-up, and analysed  *Individuals at each stage of study are described in first paragraph in Results section.* |
|  | Not applicable |  | (b) Give reasons for non-participation at each stage |
|  | Yes |  | (c) Consider use of a flow diagram  *Table 1 helps to show the study activities and input categories.* |
| Descriptive data | Yes | 14* | (a) Give characteristics of study participants (eg demographic, clinical, social) and information on exposures and potential confounders  *Table 2*: *Trachoma Impact and Surveillance Surveys Costs, Amhara, Ethiopia, 2012-2016*  *This table also includes basic descriptions of survey rounds* |
|  | Not applicable |  | (b) Indicate number of participants with missing data for each variable of interest |
| Outcome data | Yes | 15* | Report numbers of outcome events or summary measures  *Numbers and summary events are reported througouth the Results Section* |
| Main results | Yes | 16 | (*a*) Give unadjusted estimates and, if applicable, confounder-adjusted estimates and their precision (eg, 95% confidence interval). Make clear which confounders were adjusted for and why they were included  *Main results are found:*  *Table 2: Table 2*: Trachoma Impact and Surveillance Surveys Costs, Amhara, Ethiopia, 2012-2016  *Table 3: Total Costs of Trachoma Impact Surveys by Activity and Input, Amhara, Ethiopia, 2012-2016*  *Table 4: Costs by Activity and Inut as Porportion of All Costs, Amhara, Ethiopia, 2012-2016*  *Figure 1. Proportion of Total Costs by Input Catergory (for all activities) for Trachoma Impact/Surveillance Survyes, Amhara, Ethiopia, 2012-2016.*  *Figure 2. Per Cluster Costs, by Input Category for 8 Rounds of Trachoma Impact and Surveillance Surveys, Amhara, Ethiopia, 2012-2016* |
|  | Not applicable |  | (*b*) Report category boundaries when continuous variables were categorized |
|  | Not applicable |  | (*c*) If relevant, consider translating estimates of relative risk into absolute risk for a meaningful time period |
| Other analyses | Yes | 17 | Report other analyses done—eg analyses of subgroups and interactions, and sensitivity analyses  *Table 5: Drivers of Increased Cost from First 5 Rounds to Final 3 Rounds of TIS, Amhara, Ethiopia, 2012-2016* |
|  | Discussion | | |
| Key results | Yes | 18 | Summarise key results with reference to study objectives  *Results are summarized in first parapgraph in Discussion section* |
| Limitations | Yes | 19 | Discuss limitations of the study, taking into account sources of potential bias or imprecision. Discuss both direction and magnitude of any potential bias  *Discussed in pargraph 6 of Discussion* |
| Interpretation | Yes | 20 | Give a cautious overall interpretation of results considering objectives, limitations, multiplicity of analyses, results from similar studies, and other relevant evidence  *Discussed in paragpraphs 1 through 8 in Discussion section* |
| Generalisability | Yes | 21 | Discuss the generalisability (external validity) of the study results  *Discused in paragraphs 2, 3, 5 & 8 in Discussion section* |
|  | Other information | | |
| Funding | Yes | 22 | Give the source of funding and the role of the funders for the present study and, if applicable, for the original study on which the present article is based  *Discussed in Funding section* |

*Give information separately for exposed and unexposed groups.

**Note:** An Explanation and Elaboration article discusses each checklist item and gives methodological background and published examples of transparent reporting. The STROBE checklist is best used in conjunction with this article (freely available on the Web sites of PLoS Medicine at http://www.plosmedicine.org/, Annals of Internal Medicine at http://www.annals.org/, and Epidemiology at http://www.epidem.com/). Information on the STROBE Initiative is available at www.strobe-statement.org.
